# FOXA2 in cancer: from fundamental biology to clinical translation

**DOI:** 10.3389/fonc.2026.1854448

**Published:** 2026-05-28

**Authors:** Yongran Xia, Peng Liang, Lijuan Cui, Yu Cao

**Affiliations:** Department of Pathology, Suining Central Hospital, Suining, China

**Keywords:** biological functions, cancer, clinical translation prospects, FOXA2, molecular characteristics

## Abstract

Forkhead box protein A2 (FOXA2), a member of the forkhead box (FOX) transcription factor family, is essential for organ development and metabolic homeostasis. As a pioneer factor, it binds to condensed chromatin and facilitates the access of other transcription factors to DNA. Its dysregulation is a hallmark of numerous cancers, where it exhibits complex, context-dependent roles as either an oncogene or tumor suppressor, influencing these processes in a cancer type-specific manner. This review provides a comprehensive synthesis of FOXA2 biology, detailing its fundamental functions, the intricate molecular networks through which it governs malignant phenotypes across diverse tissue types, and its emerging translational potential. We systematically evaluate the promise of FOXA2 as a diagnostic and prognostic biomarker and discuss the development of novel therapeutic strategies aimed at targeting FOXA2 or its regulatory pathways. By integrating contemporary preclinical and clinical evidence, this review delineates the current landscape and future directions for leveraging FOXA2 in oncology, aiming to bridge foundational knowledge with clinical application for researchers and practitioners.

## 1 Introduction

Cancer remains the leading cause of human death globally, and its significance cannot be overstated. Identifying effective molecular targets remains a central task in tumor research. The FOX family comprises a group of transcription factors with conserved winged-helix DNA-binding domains that participate in gene transcription, cell differentiation, metabolism, and embryonic development. Notably, the FOXA subfamily, including FOXA1, FOXA2, and FOXA3, functions as pioneer factors that bind to nucleosomal DNA and locally open chromatin structures, enabling tissue-specific transcriptional programs (1–3).

FOXA2, also known as Hepatocyte Nuclear Factor 3β (HNF3β), was initially identified in liver tissue and plays a critical role in regulating glucose and lipid metabolism (4). Subsequent studies have shown that dysregulated FOXA2 expression is closely associated with the development and progression of multiple cancers, including hepatocellular carcinoma (5–10), gastric adenocarcinoma (11, 12), lung adenocarcinoma (13–22), and ovarian cancer (23–26). Depending on the cancer type, altered FOXA2 expression may either promote or suppress malignant progression and metastasis, while also influencing patient prognosis. At the molecular level, FOXA2 regulates malignant phenotypes through several mechanisms, including modulation of EMT, HIF-1α signaling, Wnt/β-catenin pathway activity, and the tumor immune microenvironment (19, 27–44). Emerging evidence also suggests that FOXA2 may participate in tumor immune escape by affecting the expression of immune checkpoint molecules such as PD-L1 (42, 45). Particularly in prostate cancer, FOXA2 significantly promotes the transition from non-NEPC to NEPC, which contributes to poor patient prognosis (46–50).

Overall, this review outlines the structure, function, and regulatory network of FOXA2, with a focus on its dual role in different cancers. Additionally, we discuss its potential for clinical translation. This review aims to provide a reference for future targeted therapy development and the exploration of diagnostic and prognostic biomarkers. Throughout this review, “FOXA2 deficiency” refers to genetic knockout or knockdown models, “reduced FOXA2 expression” refers to lower expression levels in clinical samples, and “FOXA2 loss” refers to functional loss in specific contexts.

## 2 Molecular characteristics and biological functions of FOXA2

### 2.1 Molecular characteristics

**Figure 1:**
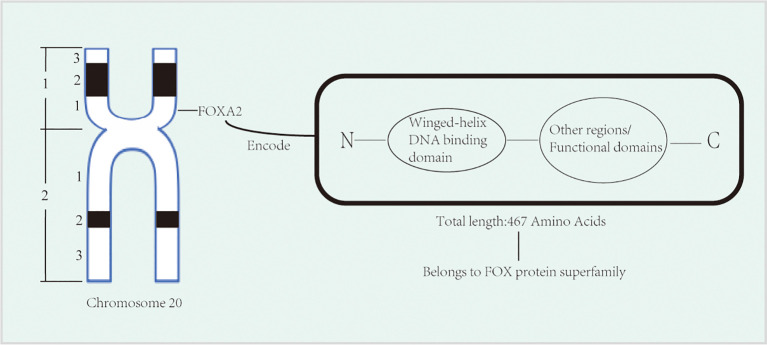
Schematic representation of FOXA2 protein structure. The FOXA2 protein contains a highly conserved winged-helix DNA-binding domain (blue) and transcriptional activation domains. As a pioneer factor, FOXA2 binds to nucleosomal DNA and opens local chromatin structures to facilitate gene transcription.

The FOXA2 gene is located on human chromosome 20p11.21 and encodes a protein composed of 467 amino acids. This protein contains a highly conserved winged-helix DNA-binding domain and belongs to the FOX protein superfamily (**Figure 1**). FOXA2 binds to tightly packed nucleosomal DNA and locally opens chromatin structures, enabling other transcription factors to access target gene loci and initiate tissue-specific expression programs.

### 2.2 Biological functions of FOXA2

**Figure 2:**
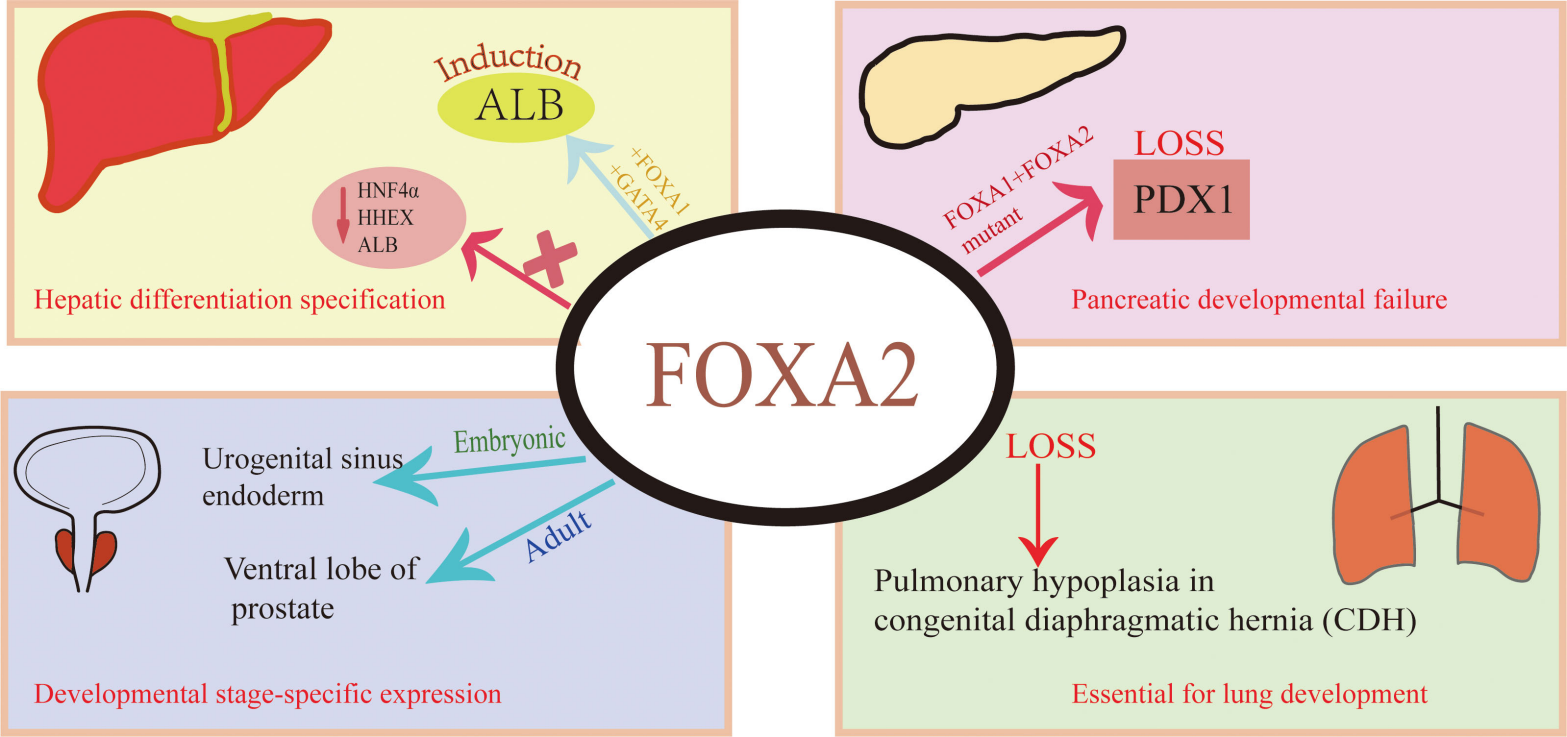
Overview of FOXA2 physiological functions. FOXA2 plays essential roles in organ development (liver, pancreas, lung, prostate) and metabolic regulation. Its dysfunction contributes to both cancerous and non-cancerous diseases.

FOXA2 plays a critical role in organ development and metabolic regulation. Its dysfunction may also contribute to non-cancer diseases (**Figure 2**).

#### 2.2.1 The role of FOXA2 in organ development

FOXA2 is broadly expressed throughout embryonic development and plays an essential role in the formation and differentiation of multiple organ systems. In the liver, loss of FOXA2 markedly impairs the expression of important developmental regulators, including HNF4α (Hepatocyte Nuclear Factor 4α) and HHEX (Hematopoietically Expressed Homeobox), as well as the differentiation marker ALB (Albumin) (51). Furthermore, FOXA1 and FOXA2 cooperate with GATA4 to induce albumin expression, thereby contributing to the initial specification of hepatic differentiation (52). During pancreatic development, FOXA2 first appears in the foregut endoderm and continues to be expressed into adulthood (53, 54). Combined mutation of FOXA1 and FOXA2 in the pancreatic anlage completely abolishes Pdx1 expression, which is considered a hallmark of normal pancreatic development, ultimately leading to developmental failure and impaired differentiation of both exocrine and endocrine cells (55). In prostate development, Mirosevich et al. systematically characterized the expression pattern of FOXA2 from embryonic to adult stages in mice and identified its specific expression within the urogenital sinus endoderm during early development and in the ventral lobe of the adult prostate (56). Similarly, during lung development, FOXA2 acts as an important etiological factor in pulmonary hypoplasia associated with congenital diaphragmatic hernia (CDH) (57).

In summary, FOXA2 serves as a master regulatory transcription factor that is indispensable for the embryogenesis and functional maturation of multiple organ systems. Its disruption leads to severe developmental defects in hepatic, pancreatic, prostate, and pulmonary tissues.

#### 2.2.2 The role of FOXA2 in metabolism

FOXA2 maintains metabolic homeostasis by binding to regulatory regions of target genes, directly activating or repressing transcription of key metabolic enzymes and transport proteins. Its activity is finely regulated by upstream signaling pathways and contributes to a complex downstream regulatory network.

##### 2.2.2.1 Glucose homeostasis

FOXA2 maintains metabolic homeostasis through its chromatin-remodeling activity by binding to regulatory regions of target genes and directly activating or repressing their transcription. In pancreatic β cells, FOXA2 is deeply involved in regulating insulin gene transcription, thereby affecting insulin production (58) and maintaining normal β cell function (59, 60). Within this complex regulatory network, the nuclear receptor FXR (Farnesoid X Receptor) acts as an upstream regulator of FOXA2. It modulates FOXA2 expression by suppressing H3K27 methylation on the FOXA2 promoter, thereby participating in the integrated control of metabolic and insulin-related gene programs (58). Intriguingly, an alternative pathway derived from pancreatic α cells also converges on FOXA2. Deficiency of FAM3A (Family with Sequence Similarity 3 Member A) in pancreatic α cells promotes GLP-1 (glucagon-like peptide-1) production, which subsequently enhances β cell function through the NR4A2-FOXA2-PC1/3 signaling axis (61). The regulatory versatility of FOXA2 underscores its essential role in maintaining systemic glucose homeostasis.

**Figure 3:**
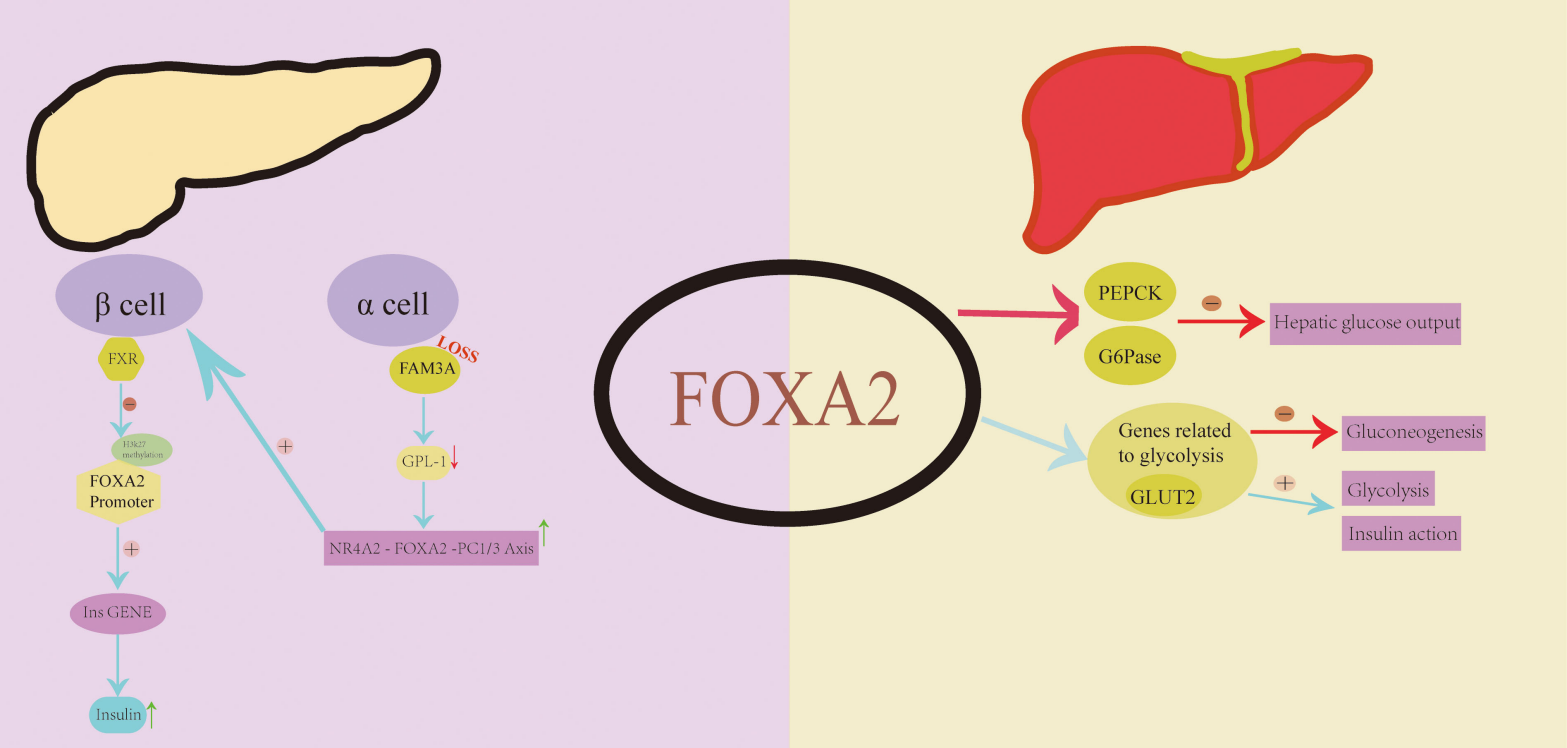
FOXA2 in hepatic glucose homeostasis. FOXA2 integrates insulin signaling and metabolic pathways to suppress gluconeogenesis (via PEPCK and G6Pase) and promote glycolysis (via GLUT2), thereby maintaining balanced hepatic glucose output.

FOXA2 integrates signals from multiple oncogenic and metabolic pathways to coordinate downstream gene expression programs (62–64). It suppresses hepatic glucose output by directly inhibiting the transcription of key gluconeogenic enzymes, phosphoenolpyruvate carboxykinase (PEPCK) (65) and glucose-6-phosphatase (G6Pase) (66). Concurrently, FOXA2 promotes glucose uptake and utilization by activating the expression of glucose transporters such as GLUT2 (67) and genes involved in glycolysis. Collectively, by coordinately inhibiting gluconeogenesis, facilitating glycolysis, and enhancing insulin action, FOXA2 acts as a critical transcriptional integrator that maintains balanced glucose metabolism in the liver (**Figure 3**).

##### 2.2.2.2 Lipid metabolism

The liver coordinates systemic lipid metabolism through lipid uptake, synthesis, oxidation, storage, and export. FOXA2 has been confirmed to play a critical role in hepatic lipid homeostasis, as its deficiency leads to hepatic steatosis and impairs the functional maturation of induced pluripotent stem cell-derived hepatocytes (68).

**Figure 4:**
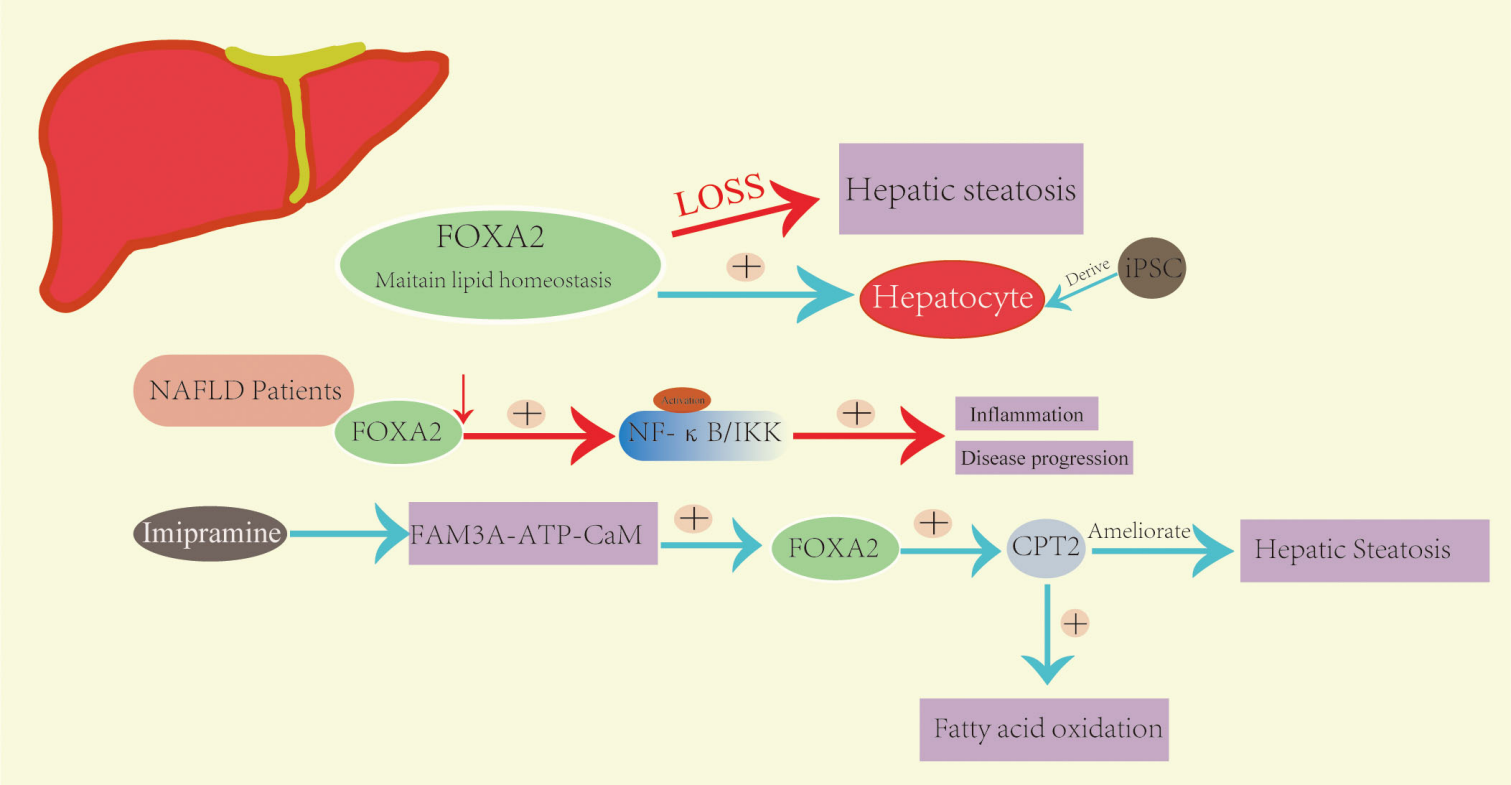
FOXA2 in NAFLD/NASH pathogenesis and therapy. Loss of FOXA2 activates NF-κB/IKK signaling, promoting inflammation and hepatic steatosis. Pharmacological agents such as imipramine enhance FOXA2 activity via the FAM3A-ATP-CaM-FOXA2-CPT2 axis, stimulating fatty acid oxidation and alleviating steatosis.

Non-alcoholic fatty liver disease (NAFLD) is one of the leading causes of liver-related mortality, with an estimated global prevalence of approximately 30% that continues to rise annually (69). In patients with NAFLD, FOXA2 expression is significantly down-regulated. Studies indicate that restoring or increasing FOXA2 levels can not only delay hepatic steatosis but also suppress activation of the NF-κB/IKK signaling pathway, thereby reducing inflammation and disease progression. These findings suggest that FOXA2 may serve as a potential therapeutic target in NAFLD through the coordinated regulation of lipid metabolism and inflammatory responses (70). In addition to endogenous regulatory pathways, such as the FXR (58) and GLP-1/NR4A2 axes (61)), exogenous pharmacological interventions can also target this network. Preclinical studies have shown that the antidepressant imipramine enhances FOXA2 activity through the FAM3A-ATP-CaM-FOXA2-CPT2 signaling cascade, thereby promoting fatty acid oxidation and alleviating hepatic steatosis in mouse models (71). Together, these findings place FOXA2 at the intersection of glucolipid metabolism and inflammation and further support its potential therapeutic relevance in NAFLD/NASH (**Figure 4**).

Given the essential role of FOXA2 in maintaining epithelial differentiation and metabolic homeostasis, its dysregulation may disrupt tissue architecture and promote malignant transformation. The following sections discuss how alterations in FOXA2 contribute to cancer development across different tissue contexts.

## 3 FOXA2 and cancer

Given FOXA2’s essential role in maintaining epithelial differentiation and metabolic homeostasis, its dysregulation may disrupt tissue architecture and facilitate malignant transformation. The following sections examine how FOXA2 alterations contribute to cancer development across diverse tissue contexts (**Table 1**).

**Table 1:** Summary of FOXA2 functions in human cancers.

Cancer type	Subtype / context	Role of FOXA2	Key signaling pathway / mechanism	Functional outcome	Ref.
Lung cancer	Small cell lung cancer (SCLC)	Oncogenic	ASCL1-FOXA2 axis	Promotes metastasis	(73)
	SCLC	Tumor suppressor	lncRNA-NEF → TGF-β1 ↓	Inhibits migration and invasion	(74)
	NSCLC	Oncogenic	MOF-mediated acetylation of SIRT6 → FOXA2 → ZEB2 ↑	Drives progression	(75)
	NSCLC	Tumor suppressor	USP54 deubiquitination → FOXA2 stabilization → ACSL4 ↑ → ferroptosis	Induces ferroptosis	(76)
	NSCLC brain metastasis	Oncogenic	FOXA2/ABAT/GABA → NF-κB ↑	Promotes brain metastasis	(77)
	Lung adenocarcinoma (KIF5B-RET)	Oncogenic	—	Regulates proliferation and invasion	(78)
	Adenosquamous carcinoma (ASC)	Involved	—	Mediates adeno-to-squamous transition	(79)
Oral cancer	Oral squamous cell carcinoma	Tumor suppressor	Binds CDH1 promoter → E-cadherin ↑	Inhibits EMT and migration	(110)
	Oral cancer	Tumor suppressor (inhibited)	MRE11/RUNX2/CXCR4/AKT → FOXA2 nuclear translocation ↓	Promotes progression (via FOXA2 cytoplasmic retention)	(111)
Esophageal cancer	ESCC	Oncogenic	FOXA2 → ZEB2 ↑	Promotes proliferation, invasion, migration	(80)
	ESCC	Oncogenic	FOXA2 → CXCR4 ↑	Enhances migration and metastasis	(81)
	Barrett’s esophagus / EAC	Oncogenic	Hedgehog → FOXA2 → MUC2 ↑	Drives squamous-to-columnar metaplasia	(82)
Gastric cancer	Gastric cancer	Tumor suppressor (induced)	miR-1291/FOXA2 axis (gambogic acid-induced)	Induces ferroptosis	(83)
	Gastric cancer	Oncogenic	HDAC3 → FOXA2 repression → FTO/m^6^A/MYC ↑	Promotes proliferation and invasion	(84)
Colorectal cancer	CRC	Oncogenic	FOXA2 → BAX	Promotes progression	(85)
	CRC (hypoxia)	Tumor suppressor	FOXA2 → hsa-let-7g → c14orf28 ↓	Inhibits hypoxia-induced EMT	(86)
	CRC	Tumor suppressor (induced)	TRIM36 → FOXA2 ↓ → NRF2/GPX4 → ferroptosis	Anti-tumor via ferroptosis	(87)
	CRC	Tumor suppressor (induced)	miR-1291/FOXA2 axis (gambogenic acid)	Inhibits proliferation and ferroptosis	(88)
	Colitis-associated CRC	Oncogenic	Neutrophil proteases/PAR2 → FOXA2-mediated autophagy dysfunction	Exacerbates colitis-to-CRC progression	(89)
	CRC	Metabolic regulator	FOXA2/ALDOB → fatty acid β-oxidation	Modulates irinotecan resistance	(90)
	CRC liver metastasis	Metabolic regulator	FOXA2 → SLC3A2 / L-glutamine metabolism	Supports colonization in liver	(91)
Pancreatic cancer	PDAC	Oncogenic	EGFR → FOXA2 → SOX9 ↑	Enhances cancer stemness and metastasis	(92)
	PNEN	Oncogenic	Methylmalonic acid → FOXA2 → INHBA ↑	Promotes progression	(93)
	Pancreatic progenitors	Tumor suppressor (loss)	FOXA2 deficiency → altered miRNA/lncRNA targeting pancreatic genes	Disrupts homeostasis	(94)
	Beta-cell differentiation	Tumor suppressor (loss)	TET1 → FOXA2-associated chromatin remodeling	Required for differentiation	(95)
	Acute pancreatitis	Protective	PIAS1 → FOXA2 sumoylation → FTO ↓	Protects against pancreatitis	(96)
Biliary tract cancer	Intrahepatic cholangiocarcinoma (ICC)	Tumor suppressor	FOXA2 loss → MAPK (p-ERK1/2, RAS, CREB1) ↑	Accelerates neoplastic changes and metastasis	(97)
Hepatocellular carcinoma	HCC	Tumor suppressor	linc00261 epigenetic silencing → FOXA2 deficiency	Promotes metastasis	(98)
	HCC	Oncogenic (migration)	O-GlcNAcylation → FOXA2 stability/activity ↑	Promotes migratory ability	(6)
	HCC (female)	Tumor suppressor	HDAC3 ablation → Foxa1/2 ↓	Promotes HCC development (gender-specific)	(100)
	HCC	Oncogenic (resistance)	—	Drives lenvatinib resistance	(8)
	HCC	Anti-apoptotic	FOXA2 → GFPT1 ↑ (HBP)	Inhibits doxorubicin-induced apoptosis	(10)
Bladder cancer	Bladder cancer	Oncogenic	EGFR → FOXA2 → SOX9 ↑	Enhances cancer stemness, invasion, metastasis	(101)
	Bladder cancer	Oncogenic	FOXA2 → GLS1 ↑ → glutamine metabolism	Promotes malignant progression	(102)
Renal cell carcinoma	Clear cell RCC	Tumor suppressor	FOXA2 → HIF1A ↓ (antagonizes VHL/HIF)	Suppresses Warburg effect and progression	(103)
			FOXA2 → glycolysis genes (GLUT1, HK2) ↓	Reduces energy supply for tumor growth	
Prostate cancer	Prostate cancer	Oncogenic	FOXA2 → AP-1 reprogramming	Drives lineage plasticity and transcriptional reprogramming	(107)
			—	Promotes bone metastasis growth	(108)
	Neuroendocrine PCa	Oncogenic	FOXA2 → lineage plasticity → KIT pathway ↑	Drives neuroendocrine transdifferentiation	(46)
			ASCL1 cooperates with FOXA2	Drives terminal neuroendocrine phenotype	(47)
			PHF8 → FOXA2 ↑ (epigenetic)	Promotes neuroendocrine progression	(49)
Cervical cancer	Cervical cancer	Oncogenic (induced)	SND1 → FOXA2 degradation ↓ → FOXA2 ↑	Promotes EMT and metastasis	(30)
	Cervical cancer	Tumor suppressor (targeted)	miR-141-3p → FOXA2 ↓	Fosters growth and invasion (via FOXA2 inhibition)	(104)
Endometrial cancer	Endometrial cancer	Tumor suppressor	FOXA2 → progesterone receptor ↑	Sensitizes to medroxyprogesterone acetate (MPA)	(106)
Ovarian cancer	Ovarian cancer	Tumor suppressor	FOXA2 → versican ↓	High FOXA2 correlates with improved survival	(23, 24)
	Ovarian cancer	Stemness regulator	FOXA2 → autophagy	Maintains cancer stem cell stemness	(25)
Thyroid cancer	Thyroid carcinoma	Tumor suppressor	FOXA2 → PKM2 ↓ → Wnt/β-catenin ↓	Blocks aerobic glycolysis	(34)
Osteosarcoma	Osteosarcoma	Tumor suppressor (induced)	lncRNA-NEF → FOXA2 → Wnt/β-catenin ↓ (baicalein-mediated)	Exerts anti-tumor activity	(35)

↑ : upregulation / increase / promotion; ↓ : downregulation / decrease / suppression; → : leads to / activates / results in.

This table lists the cancer types, subtypes/contexts, reported roles of FOXA2 (oncogenic, tumor suppressor, or context-dependent), key signaling pathways/mechanisms, functional outcomes, and corresponding references.

### 3.1 Lung cancer

**Figure 5:**
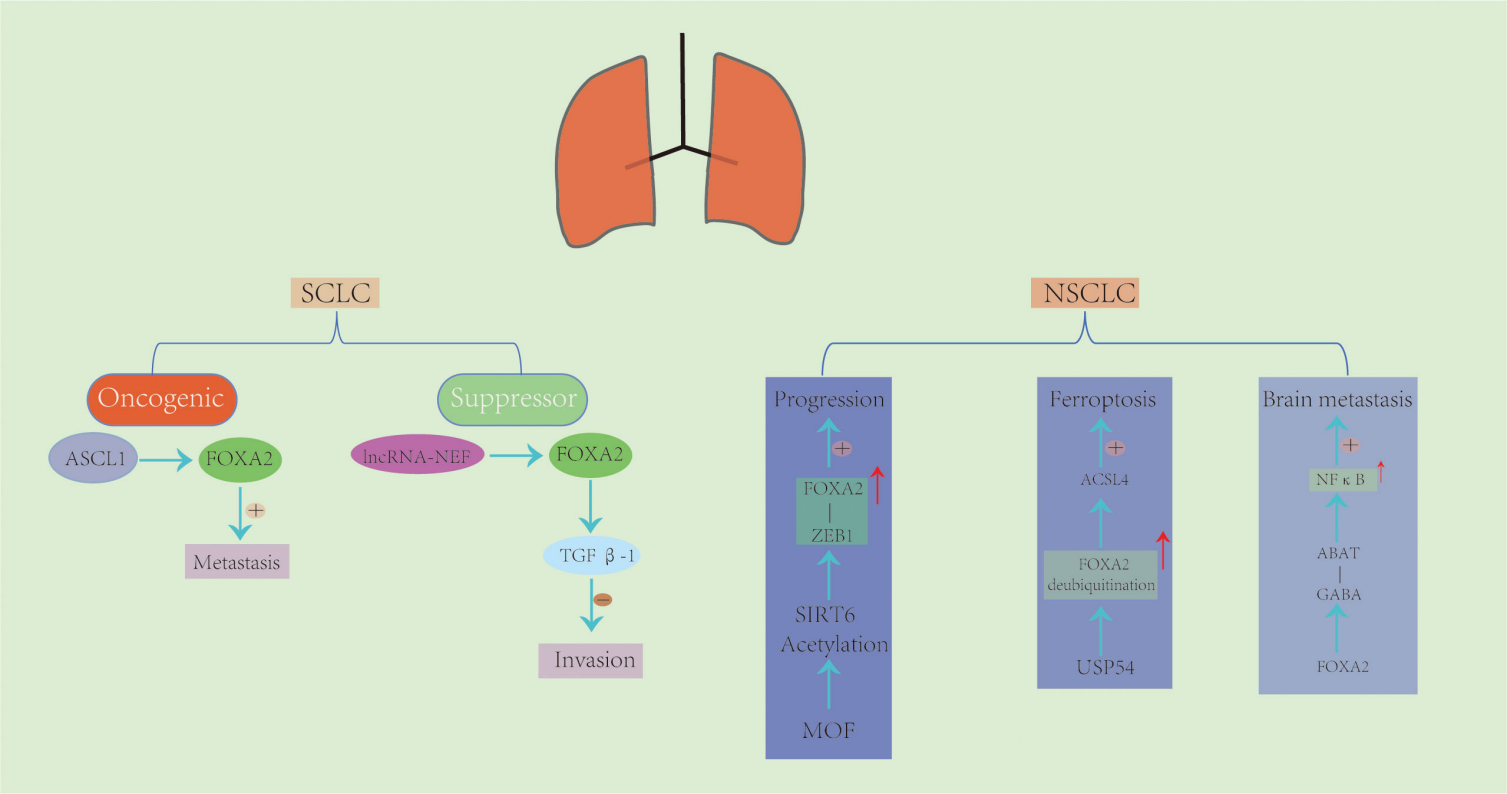
FOXA2 in lung cancer. The figure summarizes the dual roles of FOXA2 in small cell lung cancer (SCLC) and non-small cell lung cancer (NSCLC), including pro-metastatic ASCL1-FOXA2 signaling, tumor-suppressive lncRNA-NEF axis, MOF-SIRT6-mediated progression, USP54-induced ferroptosis, and FOXA2/ABAT/GABA-mediated brain metastasis.

Lung cancer is the most frequently diagnosed cancer, with an estimated 2.5 million new cases in 2022, accounting for 12.4% of total global cancer incidence. It was also the leading cause of cancer-related deaths, with an estimated 1.8 million fatalities (18.7% of total cancer deaths) (72) (**Figure 5**).

#### 3.1.1 Small cell lung cancer

Small cell lung cancer (SCLC) is a highly metastatic malignancy, which is a key factor contributing to the low survival rate of patients. Therefore, further research on therapeutic targets against SCLC metastasis holds significant importance for improving clinical treatment and prolonging patient survival. Interestingly, evidence suggests that FOXA2 exhibits context-dependent opposing effects on SCLC metastasis through different molecular axes. In the study by Kawasaki et al. (73), FOXA2 was shown to promote SCLC metastasis via the ASCL1-FOXA2 signaling axis. Conversely, the long non-coding RNA enhancer adjacent to the FOXA2 gene, lncRNA-NEF, was found to suppress SCLC metastasis (74).

This seemingly contradictory role may be attributed to cellular microenvironmental differences, epigenetic regulation, or the context-dependent specificity of signaling pathways. FOXA2’s function can be influenced by various co-regulators, epigenetic modifications, or specific cellular states, enabling it to activate pro-metastatic pathways under certain conditions while exerting tumor-suppressive effects through mechanisms such as non-coding RNA regulation in other contexts.

#### 3.1.2 Non-small cell lung cancer

Non-small cell lung cancer (NSCLC) is the most prevalent type of lung cancer. Due to limited treatment options, patients often face poor prognosis, making the exploration of targeted therapies for NSCLC imperative. Research indicates that MOF-mediated acetylation of SIRT6 impedes its interaction with FOXA2, thereby promoting the transcriptional activation of ZEB2 and driving NSCLC progression. On the other hand, USP54-mediated deubiquitination of FOXA2 reduces its degradation, stabilizes FOXA2 protein levels, subsequently enhances ACSL4 transcription, and induces ferroptosis in NSCLC cells (75).

Metastasis is a hallmark of cancer, with brain metastasis posing a significant threat to the prognosis and survival of NSCLC patients. Studies have found that gamma-aminobutyric acid (GABA) plays a key role in the development of NSCLC brain metastasis by activating the NF-κB pathway downstream of the FOXA2/ABAT/GABA axis (76).

FOXA2 also regulates rare NSCLC subtypes, including KIF5B-RET-rearranged lung adenocarcinoma and pulmonary adenosquamous carcinoma. For instance, in the KIF5B-RET gene rearrangement, which accounts for only about 1% of lung adenocarcinoma cases, FOXA2 still regulates the proliferation and invasion of these tumor cells (77). Pulmonary adenosquamous carcinoma (ASC) is a rare and aggressive NSCLC subtype with a poor prognosis. Research suggests that ASC may represent an intermediate state during the transdifferentiation from adenocarcinoma to squamous cell carcinoma, and FOXA2 is involved in this adeno-squamous transition (78). In summary, FOXA2 plays multifaceted regulatory roles in NSCLC progression, metastasis, and subtype transformation, involving mechanisms such as epigenetic modification, protein stability, and metabolic reprogramming. These findings highlight the importance of FOXA2 as a potential therapeutic target in NSCLC and underscore the complexity and context-dependency of its functions. Further elucidation of the specific regulatory networks upstream and downstream of FOXA2 in different pathways will provide crucial scientific insights for developing precise treatment strategies against NSCLC and its special subtypes.

### 3.2 Gastrointestinal tumors

#### 3.2.1 Esophageal cancer

In esophageal cancer, FOXA2 exhibits subtype-specific expression patterns and biological functions. FOXA2 is significantly upregulated in esophageal squamous cell carcinoma (ESCC) tissues and cell lines compared with normal esophageal tissues. In contrast, it is specifically expressed in Barrett’s esophagus (BE), dysplasia, and esophageal adenocarcinoma (EAC), while remaining undetectable in normal esophageal squamous epithelium. Functionally, elevated FOXA2 expression promotes the proliferation, invasion, and migration of ESCC cells, whereas FOXA2 knockdown markedly suppresses these malignant phenotypes. Mechanistically, FOXA2 promotes ESCC progression through transcriptional regulation of ZEB2 expression, and this tumor-promoting effect can be partially reversed by ZEB2 inhibition (79). Additionally, FOXA2 contributes to ESCC progression by activating CXCR4 expression, which further enhances cancer cell migration and metastatic potential (80). In the development of BE and EAC, FOXA2 acts as a downstream target of the Hedgehog signaling pathway. Gastroesophageal reflux-induced Sonic hedgehog (SHH) secretion activates Hedgehog signaling, which subsequently induces FOXA2 expression in esophageal squamous epithelial cells. FOXA2 then regulates the expression of intestinal goblet cell-related genes such as MUC2, thereby promoting squamous-to-columnar epithelial metaplasia. Clinically, reduced FOXA2 expression in ESCC tissues is closely associated with advanced TNM stage, deeper tumor invasion, lymph node metastasis, and unfavorable patient prognosis. Therefore, targeting FOXA2 or its upstream regulatory pathways may represent a potential therapeutic strategy for esophageal cancer. Suppression of FOXA2 through siRNA or small-molecule inhibitors may inhibit ESCC progression, whereas blockade of the Hedgehog signaling pathway could interfere with the FOXA2-mediated metaplastic process in BE (81).

Although substantial evidence supports the pathogenic role of FOXA2 in esophageal cancer, additional studies are still needed to clarify the molecular basis underlying its subtype-specific functional differences. Further investigations should also examine its interaction with immune checkpoint molecules and the tumor microenvironment, as well as evaluate the efficacy of FOXA2-targeted therapies in large-scale clinical studies involving different esophageal cancer subtypes.

#### 3.2.2 Gastric cancer

Gastric cancer (GC) remains one of the leading causes of cancer-related mortality worldwide, highlighting the urgent need to identify effective therapeutic targets.

Gambogic acid, a natural compound derived from Garcinia hanburyi, has been reported to exert anti-cancer effects by promoting both apoptosis and ferroptosis. Recent evidence demonstrated that gambogic acid induces ferroptosis in gastric cancer cells through the miRNA-1291/FOXA2 axis (82). Although this finding identifies a potentially actionable therapeutic pathway, several limitations should still be considered. For example, miR-1291 regulates multiple transcripts beyond FOXA2, gambogic acid exerts pleiotropic biological effects, and its therapeutic window in patients remains unclear. Therefore, future clinical translation will likely require further pharmacokinetic optimization and biomarker-based patient stratification.

Histone deacetylase 3 (HDAC3), an important regulator of epigenetic modification, can modulate the tumor-initiating activity of the FOXA2-mediated FTO/m^6^A/MYC axis in GC, thereby influencing the proliferation and invasion of gastric cancer cells (83). This mechanism further supports the important regulatory role of FOXA2 in gastric cancer pathogenesis.

Collectively, these findings indicate that FOXA2 remains an important research focus in gastric cancer and still warrants more comprehensive investigation. Future studies may further explore its role in gastric cancer initiation, progression, and clinical treatment. Potential directions include examining the dynamic changes in FOXA2 expression in tumor tissues before and after chemotherapy, as well as conducting an in-depth analysis of how FOXA2 expression status influences the development of chemotherapeutic resistance. In addition, because FOXA2 is an important transcription factor involved in human development, the mechanisms underlying its abnormal expression and regulatory alterations during gastric cancer progression deserve more systematic investigation.

FOXA2 plays an important role in development, tissue homeostasis, and disease pathogenesis. Its disease-specific expression patterns and functional alterations make it a promising candidate for clinical translation in areas such as diagnosis, prognosis, targeted therapy, and combination treatment strategies. In the following sections, we summarize the major directions, current progress, and existing challenges related to FOXA2 translational research based on recent preclinical and clinical studies.

#### 3.2.3 Colorectal cancer

FOXA2 exhibits context-dependent roles in colorectal cancer (CRC), influencing ferroptosis, autophagy, metabolic reprogramming, drug resistance, and inflammation-driven carcinogenesis. In terms of pro-tumor functions, studies suggest that FOXA2 may promote CRC progression in part by regulating BCL2-Associated X (BAX) expression, a key regulator of apoptosis, suggesting its involvement in modulating CRC cell survival (84); however, this finding requires validation *in vivo* and in independent cohorts. Moreover, FOXA2 is closely associated with the control of cellular differentiation trajectories; its dysregulation may disrupt normal differentiation processes, thereby facilitating cancer initiation and progression. Under hypoxic conditions, a common microenvironmental feature of CRC, FOXA2 transcriptionally activates hsa-let-7g, which in turn inhibits hypoxia-induced EMT by targeting c14orf28, indicating a context-specific regulatory role in CRC invasion and metastasis (85).

Regarding regulatory mechanisms of anti-tumor effects, TRIM36 suppresses FOXA2 to induce ferroptosis via the NRF2/GPX4 pathway in CRC cell lines and xenograft models (86), though the clinical relevance of this mechanism remains to be determined. Consistent with this, Gambogenic acid, a natural anti-tumor compound, inhibits CRC cell proliferation and ferroptosis via targeting two parallel axes: the miR-1291/FOXA2 axis and the AMPKα/SLC7A11/GPX4 axis, highlighting the importance of FOXA2 as a downstream target in natural product-mediated anti-CRC therapy (87).

FOXA2 is also involved in CRC-related autophagy dysfunction and colitis-associated carcinogenesis. Neutrophil-derived serine proteases can induce FOXA2-mediated autophagy dysfunction through protease-activated receptor 2 (PAR2), thereby exacerbating the progression of colitis to CRC (88). In terms of metabolic regulation, the FOXA2/ALDOB axis modulates fatty acid β-oxidation in CRC cells, and this metabolic reprogramming is closely associated with irinotecan resistance—a major challenge in CRC chemotherapy (89). Additionally, FOXA2 participates in the metabolic communication during CRC liver metastasis, with potential links to L-glutamine metabolism and the SLC3A2-mediated nutrient transport process, which supports CRC cell survival and colonization in the liver microenvironment (90).

Clinically, the dysregulation of FOXA2 and its related regulatory networks is closely correlated with CRC progression and treatment response. For instance, the FOXA2/ALDOB axis-mediated fatty acid metabolism disorder contributes to irinotecan resistance, while targeting the miR-1291/FOXA2 or AMPKα/SLC7A11/GPX4 axis may improve the efficacy of CRC treatment. Moreover, FOXA2-mediated autophagy dysfunction induced by neutrophils provides a potential link between inflammation and CRC, offering new targets for the prevention and treatment of colitis-associated CRC.

#### 3.2.4 Pancreatic cancer

In pancreatic cancer (PC), including pancreatic ductal adenocarcinoma (PDAC) and pancreatic neuroendocrine neoplasm (PNEN), FOXA2 exhibits context-dependent roles in tumor progression. It regulates PC stemness and metastasis via the EGFR/FOXA2/SOX9 axis—activated EGFR induces FOXA2, which upregulates SOX9 to enhance cancer stem cell properties, and inhibiting this axis reduces metastasis (91). Additionally, accumulation of methylmalonic acid has been reported to induce FOXA2-mediated transcriptional activation of INHBA, thereby promoting PNEN progression in experimental models (92). FOXA2 is also essential for pancreatic homeostasis: its deficiency in iPSC-derived pancreatic progenitors alters miRNA/lncRNA profiles targeting key pancreatic genes (93), and TET1 dioxygenase is required for FOXA2-associated chromatin remodeling during beta-cell differentiation, with dysfunction potentially contributing to islet dysfunction and malignant transformation (94).

FOXA2 interacts with multiple molecules to maintain pancreatic homeostasis and suppress carcinogenesis: PIAS1 protects against Cerulein-induced acute pancreatitis (a PDAC precursor) by enhancing FOXA2 sumoylation to downregulate FTO (95). Moreover, chromosomal structure alterations in PDAC cells may disrupt FOXA2 expression/function, while potential crosstalk between FOXA2 and HHEX (a core lineage commitment regulator) affects pancreatic cell plasticity (96). Overall, FOXA2’s multifaceted roles via diverse molecular mechanisms highlight its potential as a PC therapeutic target, though future studies are needed to clarify its crosstalk with key regulators, functional roles of related ncRNAs, and subtype-specific therapeutic strategies.

#### 3.2.5 Intrahepatic cholangiocarcinoma

Among biliary tract cancers (BTCs), the role of FOXA2 has been most extensively characterized in intrahepatic cholangiocarcinoma (ICC). Evidence for other BTC subtypes, such as extrahepatic cholangiocarcinoma and gallbladder carcinoma, remains limited at present. Cumulative evidence has clarified the key molecular mechanism underlying its anti-tumor effect in ICC.

FOXA2 deficiency significantly accelerates neoplastic changes in the intrahepatic bile duct, and this pro-tumorigenic effect is partially mediated by the activation of the MAPK signaling pathway. *In vivo* studies using FOXA2 knockout mice induced with ICC have shown that FOXA2 deficiency leads to prominent bile duct neoplasm formation, accompanied by significantly upregulated expression of genes involved in the MAPK signaling pathway. Key mediators of the MAPK pathway, such as p-ERK1/2, CREB1, and RAS, are highly expressed in FOXA2-deficient mice. *In vitro* experiments further confirmed that reduced FOXA2 expression exacerbates the metastatic potential of ICC cells, which is associated with the increased expression of p-ERK1/2 and RAS. Clinically, FOXA2 expression is negatively correlated with ICC tumor stage, and reduced FOXA2 expression is associated with tumor relapse and poor survival in ICC patients (97).

Collectively, this finding confirms that FOXA2 functions as a vital tumor suppressor in ICC by inhibiting the MAPK signaling pathway. The loss of FOXA2 contributes to ICC progression by activating the MAPK pathway, suggesting that FOXA2 and its related MAPK signaling pathway may serve as potential therapeutic targets for the clinical management of ICC.

Looking forward, future research could focus on three key directions: first, exploring the crosstalk between FOXA2 and other oncogenic pathways (e.g., TGF-β/Smad, PI3K/AKT) in ICC to elucidate the comprehensive regulatory network of FOXA2 in BTC progression; second, verifying the functional role of FOXA2 in other BTC subtypes (e.g., extrahepatic cholangiocarcinoma, gallbladder carcinoma) to clarify its subtype-specific tumor-suppressive mechanism; third, evaluating the clinical translation potential of FOXA2, such as developing FOXA2 expression-based prognostic stratification tools for BTC patients, and designing combination therapies targeting the MAPK pathway and FOXA2 epigenetic activators to improve the efficacy of BTC treatment.

#### 3.2.6 Hepatocellular carcinoma

In hepatocellular carcinoma (HCC), FOXA2 dysregulation disrupts its normal homeostatic functions, contributing to cell migration, metastasis, drug resistance, and apoptotic evasion. Its functional status in HCC is tightly modulated by multiple layers of regulatory mechanisms, including post-translational modification, epigenetic silencing of long non-coding RNAs (lncRNAs), and interaction with histone deacetylases (HDACs), making it a potential prognostic biomarker and therapeutic target for HCC, though the clinical relevance of this mechanism remains to be determined (98), post-translational O-GlcNAcylation enhancing protein stability and pro-migratory activity (6), and HDAC3-mediated transcriptional suppression in a sex-specific manner (99). Notably, these mechanisms yield divergent functional outcomes—O-GlcNAcylation promotes migration, whereas HDAC3 suppression promotes tumorigenesis—suggesting that the functional consequence of FOXA2 modulation depends on the specific regulatory layer affected.

FOXA2 is closely associated with HCC’s response to anti-cancer therapies, participating in both drug resistance and apoptosis regulation. Clinically, FOXA2 plays a critical role in HCC progression and lenvatinib-associated drug resistance—though the specific molecular mechanism remains to be fully elucidated, it is speculated that FOXA2 may modulate the expression of drug efflux pumps or activate oncogenic signaling pathways to reduce the sensitivity of HCC cells to lenvatinib (8). Conversely, FOXA2 exerts an anti-apoptotic effect in HCC cells under chemotherapeutic stress: it inhibits doxorubicin-induced apoptosis via transcriptionally activating GFPT1, a rate-limiting enzyme in the hexosamine biosynthetic pathway (HBP). GFPT1 activation enhances HBP flux, which may promote cell survival by regulating protein glycosylation and maintaining cellular metabolic homeostasis under doxorubicin-induced oxidative stress (10).

Collectively, FOXA2 functions as a central node in the regulatory network of HCC progression, with its expression and activity fine-tuned by O-GlcNAcylation, linc00261-mediated epigenetic silencing, and HDAC3-dependent suppression. Its dual roles in promoting metastasis and lenvatinib resistance, as well as inhibiting doxorubicin-induced apoptosis, highlight the context-dependent complexity of FOXA2 in HCC. Future research directions should focus on (1): Clarifying the detailed molecular mechanisms underlying FOXA2-mediated lenvatinib resistance (2); Exploring the therapeutic potential of targeting the linc00261-FOXA2 or HDAC3-FOXA2 axes to inhibit HCC progression; (3) Investigating the clinical value of FOXA2 as a predictive biomarker for lenvatinib or doxorubicin response, which may help optimize individualized treatment strategies for HCC patients.

### 3.3 Urological cancers

Urological cancers, including bladder cancer (BC) and renal cell carcinoma (RCC), are prevalent malignancies with poor prognosis in advanced stages. As a core member of FOX transcription factor family, FOXA2 participates in the regulation of embryonic development and tissue homeostasis, and exhibits distinct tissue-specific functional duality in urological cancers—acting either as an oncogenic driver or a tumor suppressor. Its dysregulation is closely linked to key malignant processes such as tumor metastasis, cancer stem cell (CSC) maintenance and therapeutic resistance, making it a potential prognostic biomarker and therapeutic target for urological cancer management.

#### 3.3.1 Bladder cancer

FOXA2 predominantly exerts oncogenic functions in BC, particularly in muscle-invasive subtypes. Based on key findings from a clinical and preclinical study, FOXA2 acts as a downstream effector of the EGFR signaling pathway, forming the EGFR/FOXA2/SOX9 regulatory axis to transcriptionally activate the stem cell marker SOX9. This axis enhances the self-renewal capacity of bladder cancer stem cells, thereby promoting tumor invasion and distant metastasis (100). Meanwhile, silencing FOXA2 can reduce the transcription of glutaminase 1 (GLS1), thereby inhibiting the glutamine metabolism of bladder cancer cells and suppressing the malignant progression of cancer cells (101).

#### 3.3.2 Renal cell carcinoma

FOXA2 typically functions as a canonical tumor suppressor in RCC, especially in clear cell RCC (ccRCC). A pivotal study revealed that FOXA2 directly binds to the promoter of HIF-1α to inhibit its transcription, thereby antagonizing the oncogenic VHL/HIF signaling pathway—an essential driver of ccRCC progression. Additionally, FOXA2 suppresses the Warburg effect in RCC cells by downregulating glycolysis-related genes (e.g., GLUT1, HK2), reducing the energy supply for rapid tumor growth. Clinically, reduced FOXA2 expression (as determined by immunohistochemistry) is associated with advanced TNM stage, vascular invasion, and shortened overall survival, and its deficiency contributes to resistance to tyrosine kinase inhibitors (TKIs) such as sunitinib, while FOXA2 restoration can resensitize RCC cells to TKIs (102).

### 3.4 Reproductive system

The reproductive system can be categorized into two major components based on gender: the male reproductive system and the female reproductive system.

#### 3.4.1 Female reproductive system

In cervical cancer, knockdown of staphylococcal nuclease domain-containing protein 1 (SND1) can upregulate the expression of FOXA2 by modulating the ubiquitination and degradation of FOXA2 (30). Elevated FOXA2 enhances cervical cancer cell invasiveness through EMT induction. Meanwhile, microRNA-141-3p (miR-141-3p) exerts its oncogenic function in cervical cancer primarily through targeted inhibition of FOXA2 (103).

In endometrial cancer, FOXA2 generally acts as a tumor suppressor (104), which can regulate the expression of progesterone and thereby enhance the sensitivity of cancer cells to medroxyprogesterone acetate (MPA), a commonly used oral progestin in clinical practice (105).

In ovarian cancer, high FOXA2 expression appears to be closely associated with improved patient prognosis in most studies (23, 24). Mechanistic studies have demonstrated that FOXA2 can directly bind to and regulate the expression of versican, a proteoglycan. Specifically, the expression pattern of high FOXA2-low versican in tumor tissues is positively correlated with patient survival rate (23).

In conclusion, FOXA2 exhibits distinct functional heterogeneity in different malignant tumors of the female reproductive system, and its mode of action is closely related to tumor types, providing potential molecular targets and theoretical basis for individualized targeted therapy of female reproductive system cancers.

#### 3.4.2 Male reproductive system

**Figure 6:**
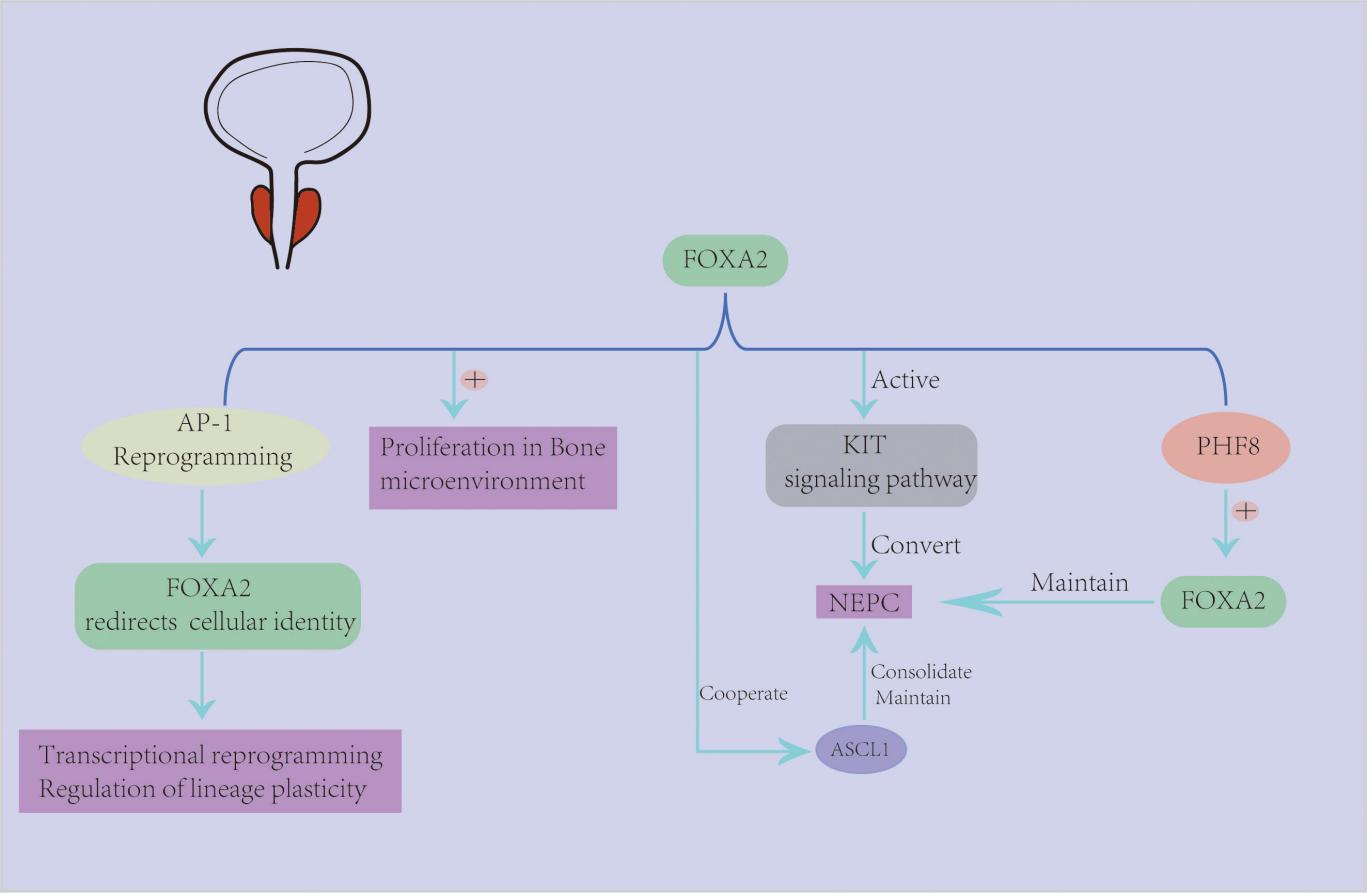
FOXA2 in prostate cancer. FOXA2 drives neuroendocrine transdifferentiation in prostate cancer through AP-1 cistrome rewiring, KIT pathway activation, and PHF8-mediated epigenetic upregulation. It also promotes bone metastasis and contributes to therapy resistance in neuroendocrine prostate cancer (NEPC).

In malignant tumors of the male reproductive system, research on the role of FOXA2 has mainly focused on prostate cancer, where it plays a crucial role in the initiation and progression of this malignancy (**Figure 6**).

FOXA2 drives pro-tumorigenic programs in prostate cancer through transcriptional reprogramming and epigenetic remodeling. One of its best-characterized core mechanisms lies in transcriptional reprogramming and the regulation of lineage plasticity via direct remodeling and functional reprogramming of AP-1 transcription factor complexes (106). By rewiring the AP-1 cistrome and altering downstream gene expression signatures, FOXA2 redirects cellular identity and promotes phenotypic switching in prostate cancer cells, a process tightly coupled with the emergence and evolution of therapy-resistant disease subtypes. Beyond regulating primary tumor progression, FOXA2 also acts as a key mediator of metastatic outgrowth, significantly promoting the proliferation and survival of prostate cancer cells in the bone microenvironment (107), which represents the most frequent and clinically devastating site of distant metastasis in advanced PCa. Established growth advantages in bone lesions further elevate tumor malignancy, accelerate disease deterioration, and markedly increases the challenges of clinical intervention. More importantly, mounting evidence has identified FOXA2 as a master driver of neuroendocrine trans-differentiation, a critical pathological event that converts conventional prostate adenocarcinoma into highly aggressive neuroendocrine prostate cancer (NEPC). Mechanistically, FOXA2 triggers profound lineage plasticity and initiates NEPC conversion through activation of the KIT signaling pathway (46), and cooperates with the core neuroendocrine transcription factor ASCL1 to consolidate and maintain the terminal neuroendocrine phenotype (47). At the epigenetic level, the histone demethylase PHF8 serves as a vital upstream regulator that epigenetically upregulates FOXA2 expression, thereby sustaining the transcriptional program necessary for neuroendocrine progression and therapy resistance (49). Together, these complementary mechanisms identify FOXA2 as a key regulatory node in prostate cancer progression, particularly in neuroendocrine transdifferentiation. In particular, FOXA2 emerges as a key molecular culprit underlying the development of lethal, treatment-refractory neuroendocrine subtypes, highlighting its great potential as a prognostic biomarker and an actionable therapeutic target for advanced and metastatic prostate cancer.

In addition to FOXA2, other molecules also affect PCa progression by regulating key pathological processes. For example, MCTP1 increases the malignant potential of castration-resistant PCa cells by inducing NE differentiation and EMT (27). NE transdifferentiation, as an important pathological event in the progression of PCa, has become a key target for clinical treatment exploration, providing direction for the development of targeted intervention strategies. Meanwhile, the application of single-cell RNA sequencing technology has revealed the existence of novel dual-negative PCa subtypes (108). The discovery of these subtypes not only enriches the subtype classification system of PCa but also provides important molecular typing evidence for in-depth analysis of the pathogenesis of different subtypes and optimization of individualized treatment plans.

In summary, mechanisms such as FOXA2-mediated transcriptional reprogramming, MCTP1-driven NE differentiation, and EMT jointly regulate the malignant progression of PCa. The discovery of novel subtypes and the therapeutic development of NE transdifferentiation drivers provide new research ideas and potential targets for improving the clinical prognosis of PCa, especially in refractory subtypes.

### 3.5 Oral cancer

FOXA2 suppresses oral cancer metastasis by transcriptionally activating CDH1 (encoding E-cadherin), thereby maintaining epithelial integrity and inhibiting cell migration (109). Reduced FOXA2 expression in oral cancer tissues is associated with lymph node metastasis and poor survival, while high FOXA2 expression correlates with elevated E-cadherin levels and favorable prognostic outcomes. Furthermore, the tumor-suppressive function of FOXA2 is tightly regulated by the MRE11/RUNX2/CXCR4/AKT signaling pathway: MRE11 promotes oral cancer progression in a nuclease-independent manner by upregulating RUNX2, which in turn increases CXCR4 expression and activates AKT, ultimately impairing FOXA2 nuclear translocation and retaining it in the cytoplasm. This regulatory axis is supported by the negative correlation between MRE11 and FOXA2 expression, as well as between FOXA2 and phosphorylated AKT, in oral cancer tissues and metastatic lymph nodes (110). The restoration of FOXA2 activity, either through PI3K/AKT inhibitors or demethylating agents targeting FOXA2, holds potential as a therapeutic strategy to suppress oral cancer metastasis and improve patient outcomes.

Despite the compelling evidence for FOXA2 as a prognostic biomarker and therapeutic target in oral cancer, further investigations are needed to elucidate the detailed epigenetic regulatory mechanisms of FOXA2 methylation, its crosstalk with other FOXA family members and tumor microenvironment, as well as to validate the efficacy of FOXA2-targeted therapies in clinical trials with larger sample sizes and diverse oral cancer subtypes.

### 3.6 Endocrine and musculoskeletal malignancies

Research on FOXA2 remains relatively scarce in tumors of other systems. Currently, it has only been clarified that in thyroid cancer, FOXA2 can inhibit the transcription of pyruvate kinase M2 (PKM2), affect the activity of the Wnt/β-catenin signaling pathway, thereby blocking the aerobic glycolysis process of thyroid cancer cells and ultimately regulating tumor initiation and progression (34). In addition, in osteosarcoma, baicalein exerts anti-tumor activity by mediating the Wnt/β-catenin signaling regulatory axis driven by lncRNA-NEF, a long non-coding RNA adjacent enhancer of FOXA2 (35). Beyond the reproductive and urinary systems, FOXA2 regulates thyroid cancer progression through PKM2-mediated glycolytic reprogramming and Wnt/β-catenin signaling (34), and suppresses osteosarcoma via lncRNA-NEF-driven modulation of the same pathway (35).

### 3.7 Controversies and conflicting evidence: the context-dependent duality of FOXA2 in cancer

FOXA2 exhibits functional duality across malignancies, acting as either a tumor suppressor or oncogene depending on cancer type, subtype, and cellular context. Conflicting results have been widely reported across studies, likely influenced by tumor subtype, disease stage, cellular model, and experimental conditions. A full appreciation of these controversies is critical for the rational clinical translation of FOXA2 as a biomarker and therapeutic target.

#### 3.7.1 Tumor suppressor vs. oncogene roles across cancer types

Current evidence supports context-dependent and cancer type-specific functions of FOXA2, which are summarized in **Table 2**.

**Table 2:** Context-dependent functions of FOXA2 in major cancer types.

Cancer type	Subtype / context	Role of FOXA2	Key mechanism / evidence	Ref.
Oral cancer	General	Tumor suppressor	Activates CDH1 (E-cadherin); MRE11/RUNX2/AKT impairs nuclear translocation	(110, 111)
Esophageal cancer	ESCC / BE / EAC	Pro-oncogenic	Activates ZEB2 and CXCR4; Hedgehog-FOXA2-MUC2 axis	(80–82)
Small cell lung cancer	Metastasis	Pro-oncogenic	ASCL1-FOXA2 signaling axis	(73)
Non-small cell lung cancer	General (progression)	Pro-oncogenic	MOF-SIRT6 → FOXA2 → ZEB2	(75)
	Ferroptosis induction	Tumor suppressor	USP54 → FOXA2 stabilization → ACSL4	(76)
Colorectal cancer	General (survival)	Pro-oncogenic	FOXA2 → BAX	(85)
	Ferroptosis induction	Tumor suppressor	TRIM36 → FOXA2 ↓ → NRF2/GPX4	(86)
Hepatocellular carcinoma	Metastasis / resistance	Predominantly tumor suppressor	FOXA2 loss due to linc00261 silencing, HDAC3 suppression, or O-GlcNAcylation alterations	(6, 8–10, 98, 100)
Intrahepatic cholangiocarcinoma	General	Tumor suppressor	FOXA2 loss → MAPK activation (p-ERK1/2, RAS, CREB1)	(97)
Prostate cancer	Neuroendocrine transdifferentiation	Pro-oncogenic	AP-1 cistrome rewiring; KIT activation; PHF8-mediated upregulation	(46, 47, 49, 107, 108)
Renal cell carcinoma	Clear cell RCC	Tumor suppressor	FOXA2 → HIF1A ↓, glycolysis genes ↓	(103)
Ovarian cancer	General	Tumor suppressor	Regulates versican expression	(23, 24)

This table contrasts the tumor-suppressive versus pro-oncogenic roles of FOXA2 across selected cancer types, summarizing the key mechanisms and supporting evidence.

#### 3.7.2 Mechanisms underlying functional duality

The opposing roles of FOXA2 can be explained by five mechanisms that are not mutually exclusive:

Cellular context and co-factor availability. FOXA2 opens chromatin but depends on cell-specific co-regulators to determine transcriptional output. It cooperates with ASCL1 in prostate cancer neuroendocrine differentiation (47) but interacts with distinct partners in hepatocellular carcinoma to suppress metastasis (9).

Post-translational modifications (PTMs). FOXA2 activity is modulated by phosphorylation, acetylation, O-GlcNAcylation, and sumoylation. These modifications alter subcellular localization, DNA-binding affinity, and protein stability. In HCC, O-GlcNAcylation stabilizes FOXA2 and promotes migration (6), whereas other PTM patterns may favor tumor-suppressive functions.

Tumor microenvironment (TME). Hypoxia, inflammatory cytokines, and stromal components vary considerably across cancer types and within individual tumors. FOXA2 regulates HIF signaling (27, 31, 33) and interacts with immune checkpoint pathways (42, 45), indicating that TME composition strongly shapes its net biological function.

Epigenetic regulation. The FOXA2 locus undergoes cancer-specific epigenetic silencing or activation via promoter hypermethylation or lncRNA-mediated repression (e.g., linc00261 in HCC, lncRNA-NEF in SCLC), leading to dramatic changes in expression and functional switching (13, 74, 99).

Redundancy and crosstalk with FOXA family members. FOXA1 and FOXA3 share high sequence homology with FOXA2 and are often co-expressed. Loss of FOXA2 may be compensated by FOXA1 in some contexts, whereas the two factors exert opposing effects in others (15–18). This complex interplay remains incompletely understood.

#### 3.7.3 Methodological limitations of current studies

When interpreting conflicting reports, several key limitations should be acknowledged:

Model system discrepancies. Established cancer cell lines often do not faithfully recapitulate tumor heterogeneity or the tumor microenvironment; *in vitro* findings may not translate to *in vivo* models or patient tissues.

Antibody specificity. High conservation among FOXA proteins may cause cross-reactivity; IHC−based expression data require rigorous antibody validation.

Sample size and statistical power. Studies with small clinical cohorts (<50-100 cases) are prone to false-positive or false-negative associations.

Publication bias. Positive or novel results are more likely to be published, potentially skewing the perceived strength of evidence.

#### 3.7.4 Outstanding questions

Future investigations should address the following unresolved issues:

1. What molecular switches determine whether FOXA2 functions as an oncogene or tumor suppressor in a given cellular context?
2. Can post-translational modifications of FOXA2 be therapeutically targeted to redirect its functional output?
3. How does the FOXA1-FOXA2-FOXA3 regulatory network operate in cancers where all three factors are co-expressed?
4. What biomarkers can reliably predict tumor responses to FOXA2-targeted therapies?
5. How does FOXA2 shape the tumor immune microenvironment, and can it be targeted to enhance the efficacy of immune checkpoint inhibitors?

#### 3.7.5 Integrative perspective

The context-dependent duality of FOXA2 can be conceptualized through a “pioneer factor availability” model. In cancers where FOXA2 maintains differentiation programs (e.g., HCC, ICC, RCC), its loss de-differentiates cells toward a progenitor-like, aggressive state. Conversely, in cancers where FOXA2 drives transdifferentiation (e.g., prostate NEPC, esophageal SCC), its gain hijacks developmental programs for malignant transformation. This model predicts that FOXA2 function depends on (1) the differentiation state of the cell of origin, (2) the availability of lineage-specific co-factors, and (3) the epigenetic landscape shaped by prior oncogenic events. Testing this model will require lineage-tracing studies in genetically engineered mouse models and single-cell multi-omics in human tumors.

## 4 Clinical translation prospects of FOXA2

FOXA2 plays a central role in development, homeostasis, and disease pathogenesis. Its disease-specific expression and functional changes make it a promising target for clinical translation, including diagnosis, prognosis, targeted therapy, and combination regimens. Below, we will summarize several major directions, progress, and existing issues related to FOXA2 translation, based on recent preclinical and clinical research.

### 4.1 FOXA2 as a molecular biomarker for disease diagnosis and prognosis evaluation

Reliable molecular markers are essential for early intervention, prognostic stratification, and individualized treatment. FOXA2 has a relatively specific expression pattern in various diseases, and its expression level is closely related to disease progression and clinical outcomes. Therefore, it has the potential to become a promising diagnostic/prognostic marker, and there is some evidence supporting this in both oncology and metabolic diseases.

#### 4.1.1 Diagnostic and prognostic value in cancer

The expression characteristics of FOXA2 vary significantly across different cancer types, but most are associated with prognosis. Some studies have also confirmed its potential to predict recurrence. For example, in small cell lung cancer (SCLC), clinical cohort studies have found that 61% of patients have multiple organ metastasis at diagnosis, and the expression of FOXA2 in metastasis-related samples is significantly higher than in non-metastasis samples. Further tissue microarray analysis also verified that SCLC patients with high FOXA2 expression have a significantly higher recurrence rate and a significantly shorter recurrence-free survival (73). This indicates that FOXA2 can serve as a specific biomarker for assessing the recurrence risk of SCLC, providing valuable reference for optimizing treatment plans.

In addition to prognostic assessment, FOXA2 can also assist in tumor molecular typing and treatment sensitivity prediction. Professor Wang Jianliu’s team identified 17 prognostic genes related to progesterone receptor (PR) status in endometrial cancer through transcriptome analysis, and constructed a molecular typing label that can accurately distinguish between patients with high and low PR expression (105). As a key upstream transcriptional regulator of PR, the expression level of FOXA2 is significantly positively correlated with PR, which indirectly suggests that patients with high FOXA2 expression may be more likely to benefit from progesterone treatment - this is an important reference for the strategic choice of fertility-preserving treatment for premenopausal patients with endometrial cancer.

Furthermore, FOXA2 gene mutation detection has entered clinical application. Commercially available FOXA2 gene testing panels can detect sequence variants and copy number alterations with reported high sensitivity.

#### 4.1.2 Diagnostic potential in non-cancerous diseases

In the field of metabolic diseases, FOXA2 also demonstrates diagnostic value. Clinical data have shown that FOXA2 gene polymorphism (e.g., rs1209523) can reduce the risk of type 2 diabetes mellitus by 25-30% (111). Detection of this polymorphic locus can be used as a screening indicator for populations at high risk of type 2 diabetes mellitus, laying a foundation for early disease prevention. Furthermore, FOXA2 plays a central role in pancreatic islet β-cell development and glucose homeostasis regulation. HiPSC model studies confirmed that FOXA2 expression silencing can lead to a 70% decrease in β-cell proliferation capacity (93). Since β-cell dysfunction is a core pathological feature of type 2 diabetes mellitus, FOXA2 expression level may serve as a potential molecular indicator for evaluating islet function, assisting in the early diagnosis of diabetes mellitus.

### 4.2 Development of FOXA2-targeted therapeutic strategies

Given the pivotal regulatory role of FOXA2 in disease pathogenesis, therapeutic strategies targeting it directly and its upstream and downstream regulatory axes have emerged as a research hotspot. These strategies encompass various directions, including small molecule drugs, gene intervention, metabolic intervention, and combination therapy. Some of these strategies have already progressed to the preclinical validation stage, indicating promising prospects.

#### 4.2.1 Small molecule drug targeted therapy

Small molecule drugs, due to their convenient administration and relatively strong targeting ability, are the main direction of FOXA2 targeted therapy.

In cancer therapy, small-molecule modulators of FOXA2 upstream pathways have shown preclinical efficacy. Taking endometrial cancer as an example, the FDA-approved fatty acid synthase inhibitor Orlistat can reduce the phosphorylation of FOXA2 at Thr156 indirectly by modulating AKT activity (this phosphorylation modification promotes FOXA2 degradation), thereby stabilizing FOXA2 protein levels and further upregulating PR expression, reversing progesterone resistance in tumor cells. Both *in vitro* experiments and animal models have confirmed that Orlistat combined with progestins (such as medroxyprogesterone acetate, MPA) can significantly inhibit tumor growth, with better effects than either drug alone (105). This combination regimen has entered the phase of prospective clinical trials.

#### 4.2.2 Gene intervention therapy

Gene intervention technology directly regulates FOXA2 expression, correcting its functional abnormalities and offering unique advantages in disease treatment. In tumor therapy, gene knockdown strategies have also demonstrated clear anti-metastatic effects: in SCLC cell lines and patient-derived xenograft (PDX) models, lentivirus-mediated shRNA knockdown of FOXA2 significantly reduces the number of metastatic foci in multiple organs such as the liver and adrenal glands, decreasing metastatic burden without affecting the proliferative capacity of tumor cells (73). This indicates that targeting FOXA2 gene knockdown can serve as a precise therapeutic strategy for inhibiting SCLC metastasis.

#### 4.2.3 Metabolic intervention and combination therapy strategies

The close association between FOXA2 and metabolic pathways provides a potential direction for metabolic intervention in disease treatment, and combining metabolic intervention with conventional therapies may further improve therapeutic efficacy. In addition to the previously mentioned Orlistat combined with progestogen regimen, recent studies have established a novel comprehensive metabolic intervention strategy termed “one-anti, three-lowering,” which involves estrogen suppression combined with targeted reductions in blood glucose, calcium, and lipid levels during fertility-preserving treatment for endometrial cancer. By modulating the metabolic state within the tumor microenvironment, this strategy indirectly affects FOXA2 expression and function, which significantly improves the complete response rate. Notably, an international multicenter randomized controlled trial evaluating metformin combined with progestogen demonstrated that metformin can significantly improve the complete response rate in patients with atypical hyperplasia (112). This regimen has subsequently been incorporated into clinical guidelines in several countries.

In comprehensive cancer treatment, the potential combination of FOXA2-targeted strategies with chemotherapy and immunotherapy also deserves further attention. Since FOXA2 mainly regulates tumor metastatic capacity rather than proliferative activity, combining FOXA2-targeted approaches with chemotherapeutic agents such as paclitaxel and cisplatin may help inhibit metastatic progression while simultaneously suppressing tumor growth, ultimately improving the prognosis of patients with advanced cancer (113, 114). In addition, the role of FOXA2 in regulating the tumor immune microenvironment is still being explored, and future combination with immune checkpoint inhibitors may further enhance anti-tumor efficacy.

### 4.3 Challenges and countermeasures in FOXA2 clinical translation

Despite its promising translational potential, FOXA2-targeted strategies face challenges related to target specificity, model fidelity, and limited clinical validation. These challenges require gradual resolution through multidisciplinary collaboration.

#### 4.3.1 Core challenges

The first issue is insufficient targeting specificity. Members of the FOXA family (FOXA1, FOXA2, etc.) share high homology and exhibit either synergistic or antagonistic relationships in terms of function. Most small molecule drugs currently developed that target FOXA2-related pathways lack sufficient specificity for individual FOXA family members, potentially leading to off-target effects. The second issue concerns phenotypic differences between animal models and human diseases. For instance, the mortality rate resulting from FOXA2 knockout in mice does not align with human scenarios, and some treatment strategies that are effective in preclinical settings may not perform well in human trials, thereby affecting translational efficiency. The third issue is the lack of clinical research evidence. Currently, most studies are still in the preclinical phase, lacking large-sample, multicenter, prospective clinical trials for validation. Furthermore, FOXA2 exhibits significant variations in expression and function across different disease subtypes and patient populations, complicating the development of individualized clinical protocols. The fourth issue pertains to the limitations of detection technology. Existing FOXA2 detection methods (such as NGS) struggle to accurately detect genomic changes like single exon deletions/duplications and variations in the transcriptional regulatory regions, potentially leading to the omission of some pathogenic variations.

#### 4.3.2 Countermeasures

Addressing the challenges described above requires a multifaceted approach that includes molecular mechanism research, technology development, and clinical study design. At the molecular level, multi-omics technologies such as single-cell ATAC-seq and spatial transcriptomics should be applied to accurately characterize the FOXA2 regulatory network across different diseases and cell types. These approaches may also help clarify the functional differences between FOXA2 and other FOXA family members, thereby providing a theoretical basis for the development of highly specific therapeutic agents. At the technology development level, detection methods should be further optimized to improve the sensitivity for identifying rare variations. At the same time, condition-specific regulatory systems, including cell-specific promoter-mediated gene regulation and light-controlled small-molecule drugs, should be developed to improve treatment specificity and safety. At the clinical research level, stronger multi-center collaboration and large-scale prospective clinical trials are still needed. Integrating molecular typing technologies may further support precise patient stratification and improve the clinical relevance of treatment strategies. Additionally, establishing animal models that more closely resemble human disease conditions, such as humanized PDX models and gene-edited mouse models, may further enhance the translational value of preclinical findings.

Overall, FOXA2 has emerged as a promising target for clinical translation because of its central regulatory role in disease pathogenesis. As research into its molecular mechanisms continues to advance, together with ongoing improvements in targeted therapies and gene intervention technologies, FOXA2-related diagnostic and therapeutic strategies are expected to provide new opportunities for the precise prevention and treatment of multiple diseases and may further promote innovation in clinical management strategies.

## 5 Discussion and perspective

### 5.1 Summary of core conclusions

FOXA2 coordinates chromatin remodeling and gene expression regulation through its conserved DNA-binding domain and transcriptional activation domains, with expression patterns exhibiting tissue and developmental stage specificity. At the regulatory mechanism level, the expression and activity of FOXA2 are finely regulated by multiple factors, including upstream transcription factors, non-coding RNA epigenetic modifications, and post-translational modifications (phosphorylation, acetylation, etc.).

In a physiological state, FOXA2 serves as a core regulatory factor in embryonic endoderm differentiation and organ formation (such as liver, pancreas, and lung), and plays an irreplaceable role in maintaining tissue homeostasis in adults, particularly in regulating glucose and lipid metabolism balance and pancreatic β-cell function. When FOXA2 expression or function becomes abnormal, its dual role in pathological processes gradually emerges. In the field of oncology, FOXA2 exhibits a clear context-dependent duality as both an oncogene and a tumor suppressor, with its function closely linked to cancer type, tumor stage, and microenvironment. As systematically summarized in Section 3.7, FOXA2 acts as a tumor suppressor in oral cancer, intrahepatic cholangiocarcinoma, and renal cell carcinoma, but exerts pro-oncogenic effects in esophageal cancer, small cell lung cancer, and prostate cancer. For example, high expression of FOXA2 in small cell lung cancer promotes tumor metastasis, whereas FOXA2 functions as a tumor suppressor in intrahepatic cholangiocarcinoma and renal cell carcinoma. In non-tumor diseases, abnormal FOXA2 function is closely related to the occurrence and development of type 2 diabetes, obesity, and cardiovascular diseases, and its gene polymorphism has become an important molecular marker for risk assessment of metabolic diseases.

In terms of clinical translation, FOXA2 exhibits dual application value: on the one hand, its expression level and gene polymorphism can serve as novel molecular markers for tumor prognostic stratification, chemotherapy sensitivity prediction, and metabolic disease risk screening; on the other hand, therapeutic strategies targeting FOXA2 and its regulatory pathways (such as small molecule inhibitors, gene intervention, and metabolic combination therapy) have achieved breakthroughs in preclinical research. Some combination regimens (such as Orlistat combined with progesterone for the treatment of endometrial cancer) have entered the clinical trial stage, providing a new direction for precision medicine.

### 5.2 Existing research limitations

Although considerable progress has been made in the research on FOXA2, many scientific questions remain unresolved. In addition to the methodological limitations noted in Section 3.7.3—model system discrepancies, antibody cross-reactivity, insufficient sample sizes, and publication bias—several fundamental knowledge gaps remain.

Firstly, the molecular mechanism underlying the functional heterogeneity of FOXA2 remains incompletely understood. FOXA2 exerts completely opposite effects in different cancer subtypes and even in different stages of the same tumor. While Section 3.7.2 proposes five potential mechanisms (cellular context, post-translational modifications, tumor microenvironment, epigenetic regulation, and FOXA family redundancy), the precise molecular switches that determine whether FOXA2 functions as an oncogene or tumor suppressor in a given context have not been systematically elucidated. This greatly limits its clinical application as a target for targeted therapy.

Secondly, the distinction between functional redundancy and specificity among members of the FOXA family is not sufficient. The DNA-binding domains of FOXA1, FOXA2, and FOXA3 are highly homologous and co-expressed in multiple tissues. Current research struggles to distinguish the independent functions of individual family members, potentially leading to off-target effects during the development of targeted drugs and affecting treatment safety.

Thirdly, the role of FOXA2 in the non-coding RNA regulatory network still requires further exploration. Currently, most research on FOXA2 regulation focuses on the protein level, with limited studies on the targeted regulatory mechanisms of long non-coding RNA and circular RNA on FOXA2, as well as the bidirectional regulatory network in which FOXA2, as a transcription factor, regulates the expression of non-coding RNA.

Fourthly, there are translational barriers in clinical translational research. Most FOXA2 targeted therapeutic strategies are still at the stage of cell lines and mouse models. Due to the differences in pathophysiological characteristics between animal models and human diseases, some effective protocols in animal experiments may not perform well in clinical trials. At the same time, there is a lack of large-sample, multicenter prospective cohort studies to verify the clinical value of FOXA2 as a molecular marker, which limits its clinical application and promotion.

### 5.3 Future research directions

#### 5.3.1 Defining molecular switches of FOXA2 functional duality

Single-cell multi-omics (scRNA-seq, scATAC-seq, spatial transcriptomics) should be applied to map FOXA2 expression, chromatin occupancy, and co-factor recruitment across tumor subclones and microenvironmental niches. Priority should be given to prostate cancer (adenocarcinoma-to-NEPC transition) and lung cancer (adenocarcinoma-to-squamous transdifferentiation), where FOXA2 undergoes documented functional switching.

#### 5.3.2 Exploring the functional specificity and synergistic mechanisms of FOXA family members

Animal models with specific knockout/knock-in of FOXA family members can be established using gene editing technology. Combined with protein interactomics (e.g., Co-IP, mass spectrometry analysis), the interaction patterns and functional division of labor among different members can be clarified. Small molecule inhibitors targeting the specific structural domains of FOXA2 can be developed to reduce cross-reactivity with other family members and improve the specificity and safety of targeted therapy.

#### 5.3.3 Expanding clinical translation research of FOXA2 in multiple disease fields

On the one hand, efforts should be made to promote clinical validation of FOXA2 as a molecular biomarker, conducting large-sample prospective cohort studies to determine its cutoff values and clinical application criteria for cancer prognostic evaluation and treatment sensitivity prediction. On the other hand, FOXA2-targeted combination therapy strategies should be optimized, such as combining FOXA2-targeted drugs with novel therapeutic approaches like immune checkpoint inhibitors and oncolytic viruses to explore synergistic enhancement mechanisms. Meanwhile, FOXA2-based gene therapy delivery systems (e.g., tumor-targeted adenoviral vectors, lipid nanoparticles) should be developed to improve the targeting and efficacy of gene intervention.

#### 5.3.4 Uncovering the potential value of FOXA2 in combined disease prevention and control

Given the dual regulatory role of FOXA2 in metabolic and tumor fields, future research can explore the interdisciplinary direction of “metabolic intervention-tumor prevention”. For example, investigating the mechanism by which FOXA2-regulated dietary interventions and exercise therapies reduce the risk of obesity-related cancers (e.g., liver cancer, colorectal cancer) can provide new ideas for achieving combined prevention and control of metabolic diseases and cancers.

### 5.4 Concluding remarks

In summary, as a core molecular hub connecting physiological homeostasis and pathological dysregulation, FOXA2 research not only deepens the mechanistic understanding of disease development but also holds great promise for advancing novel diagnostic biomarkers and targeted therapeutics. The context-dependent functional duality systematically highlighted in this review emphasizes the critical need to account for cancer type, disease stage, and tumor microenvironment when interpreting experimental data and designing clinical applications. With ongoing technological advances and deeper mechanistic insights, FOXA2 will undoubtedly become an increasingly important molecule in the era of precision oncology.
